# The role of interleukin-33 in organ fibrosis

**DOI:** 10.1093/discim/kyac006

**Published:** 2022-09-26

**Authors:** Samuele Di Carmine, Molly M Scott, Mairi H McLean, Henry J McSorley

**Affiliations:** Division of Cell Signalling and Immunology, School of Life Sciences, Wellcome Trust Building, University of Dundee, Dundee, UK; Division of Molecular and Clinical Medicine, School of Medicine, University of Dundee, Ninewells Hospital, Dundee, UK; Division of Molecular and Clinical Medicine, School of Medicine, University of Dundee, Ninewells Hospital, Dundee, UK; Division of Cell Signalling and Immunology, School of Life Sciences, Wellcome Trust Building, University of Dundee, Dundee, UK

**Keywords:** IL-33, fibrosis, acute injury, chronic injury, wound healing

## Abstract

Interleukin (IL)-33 is highly expressed in the nucleus of cells present at barrier sites and signals via the ST2 receptor. IL-33 signalling via ST2 is essential for return to tissue homeostasis after acute inflammation, promoting fibrinogenesis and wound healing at injury sites. However, this wound-healing response becomes aberrant during chronic or sustained inflammation, leading to transforming growth factor beta (TGF-β) release, excessive extracellular matrix deposition, and fibrosis. This review addresses the role of the IL-33 pathway in fibrotic diseases of the lung, liver, gastrointestinal tract, skin, kidney and heart. In the lung and liver, IL-33 release leads to the activation of pro-fibrotic TGF-β, and in these sites, IL-33 has clear pro-fibrotic roles. In the gastrointestinal tract, skin, and kidney, the role of IL-33 is more complex, being both pro-fibrotic and tissue protective. Finally, in the heart, IL-33 serves cardioprotective functions by favouring tissue healing and preventing cardiomyocyte death. Altogether, this review indicates the presence of an unclear and delicate balance between resolving and pro-fibrotic capabilities of IL-33, which has a central role in the modulation of type 2 inflammation and fibrosis in response to tissue injury.

## Graphical Abstract

**Graphical Abstract:**
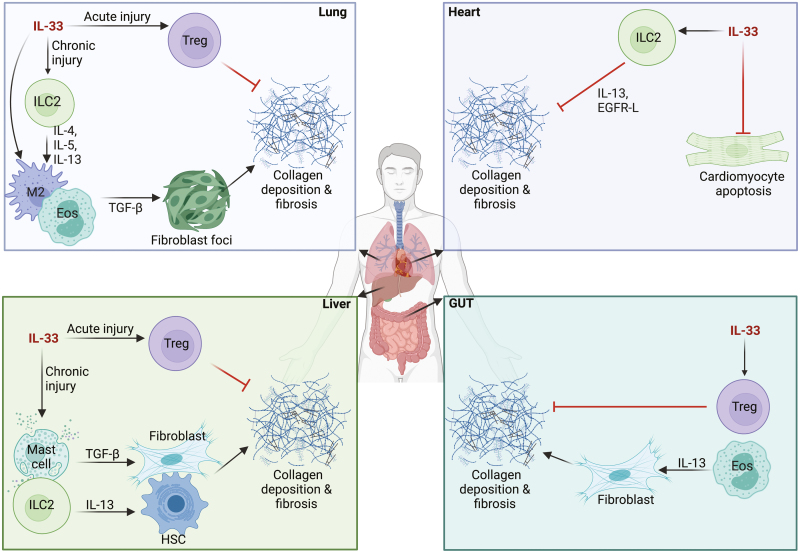

## Introduction to fibrosis

Fibrosis arises from an abnormal deposition of scar tissue at the site of tissue injury or inflammation. Normal responses to injury include the wound-healing response, which comprises four stages: haemostasis or coagulation, inflammation, proliferation, and remodelling with resolution. Fibrosis arises from chronic inflammation and is characterized by a dysregulated wound-healing phase. Aberrant wound healing causes sustained proliferation and remodelling of the extracellular matrix (ECM) and collagen deposition at injury sites. This process leads to the formation of fibrotic scar tissue [1], comprising structural scaffolding fibrillar collagens I and III, collagen IV-anchored laminin and fibronectin in the basement membrane, and elastin [2]. Organ fibrosis is associated with significant morbidity and mortality. Therefore, there is a clinical need to understand the biological mechanisms of fibrosis to identify new treatment targets and biomarkers to aid diagnosis, measure treatment response, and predict prognosis.

Following haemostasis and clotting of the wound, damaged stromal cells and coagulated platelets release damage-associated molecular patterns; alarmins such as IL-1α and IL-33; and inflammatory chemokines, which recruit immune cells and lead to the inflammatory phase of injury response [3].

One of the first events in the inflammatory phase of the injury response is the recruitment of phagocytic immune cells such as monocyte/macrophages and neutrophils. Recruited monocytes differentiate into macrophages, which are then polarized towards the pro-inflammatory M1 phenotype by interferon gamma (IFN-γ), tumour necrosis factor alpha (TNF-α), and bacterial or viral molecules at the injury site. Here they produce pro-inflammatory cytokines IL-1β, TNF-α, IL-12, IL-18, and IL-36, all of which are elevated in fibrotic disease [4, 5]. In particular, IL-36 can stimulate inflammatory and epithelial cells to activate the nuclear factor-κB (NF-κB) pathway in a positive feedback loop, thus exacerbating pro-fibrotic immune responses [6]. M1 macrophages and neutrophils are effective in the killing and clearance of invading microorganisms, required to allow resolution of inflammation. In cases of prolonged inflammation, excessive recruitment of neutrophils prevents the resolution of inflammation, thus creating the aberrant wound healing seen in fibrosis [7].

As the wound-healing response progresses towards proliferation and remodelling phases, IL-4 and IL-13 are released, inducing a switch from M1 to M2 macrophage differentiation, further aided by IL-37 expressed by myeloid lineage cells [8, 9]. M2 macrophages secrete matrix metalloprotease-9 (MMP-9), IL-10, and transforming growth factor beta (TGF-β) and result in ECM remodelling, reduction of inflammation, and proliferation and activation of fibroblasts [7, 10]. Recent findings suggest that the M1/M2 paradigm of macrophage polarization is an oversimplification, and at least four subtypes of M2 macrophage responsible for the signalling which guide the wound-healing response through to conclusion, of which type M2a, induced by IL-4 and IL-13, is responsible for recruitment and activation of fibroblasts and produce collagen precursors [11]. Persistent inflammation is likely to prevent the signalling that should cause the progression through the M2 subtypes and conclude the wound-healing response. Instead, the effects of M1 and M2a macrophage signalling continue to promote inflammation, ECM synthesis, and secretion in fibroblasts, leading to fibrosis [10]..

Recruited neutrophils contribute to wound healing through the secretion of elastase and MMPs [12]. Eosinophils are also recruited during the wound-healing response. In haemostasis, IL-5 activated eosinophils process fibrinogen into fibrin to contribute to coagulation of the wound, and exposure to fibrinogen can trigger degranulation in eosinophils. This has been shown to cause the secretion of pro-inflammatory factors from pulmonary epithelial cells such as TGF-α, TGF-β, platelet-derived growth factor (PDGF), and MMP-9 and increases TGF-β secretion in fibroblasts [13–15]. These cytokines are implicated in the proliferative and remodelling stages of the wound-healing response, as are the cytokines released directly from eosinophils: TGF-β, IL-1β, and TNF-α. Eosinophils also release IL-4 and IL-13, further polarizing towards type 2 immunity and activation of fibroblasts [16, 17].

Overall, these cell responses in fibrosis are orchestrated by cytokines such as IL-4 and IL-13, which induce M2 macrophage differentiation, and IL-5, which recruits eosinophils [18]. A potent source of these cytokines is the group 2 innate lymphoid cells (ILC2s), which stimulate and maintain the wound-healing response [19, 20]. ILC2s are innate lymphocytes, and their activation and cytokine release are stimulated by the epithelial cytokines IL-25, IL-33, and TSLP (thymic stromal lymphopoietin).

The common cellular pathway that all of these immune responses lead to is the recruitment, activation, and proliferation of fibroblasts. Fibroblasts are responsible for contraction of the wound site and the synthesis and release of ECM proteins. Fibroblasts have high expression of α-smooth muscle actin (α-SMA) and mesenchymal marker vimentin [21]. They migrate to the wound site following exposure to plasma fibronectin, which is deposited alongside cellular fibronectin released from platelets [22]. IL-10, IL-33, PDGF, and TGF-β are key regulators of fibroblast recruitment and activation, alongside IL-4 and IL-13, which promote the synthesis and deposition of ECM proteins such as collagen, fibronectin, and elastin [4, 23, 24]. These cytokines also cause the differentiation of fibroblasts into myofibroblasts, which are responsible for the mechanical wound contraction facilitated by α-SMA. The ECM is critical for re-epithelization as it forms the basement membrane for the new epithelial cells to attach to and influences the polarization of epithelial cells, which is essential for their function [25]. Overstimulation and failure to downregulate fibroblast activity leads to excessive ECM deposition, which results in fibrosis, containing fibrillar proteins such as elastin and collagen I and III, and non-fibrillar proteins like fibronectin. Fibrillar proteins form filaments and make up structural scaffolds, whereas non-fibrillar proteins form the basement membrane, proteoglycans, and glycoproteins [26].

MMPs control ECM deposition and breakdown by degrading excessive ECM, while tissue inhibitors of metalloproteinases (TIMPs) downregulate MMP activity, promoting ECM accumulation [1]. While upregulation of TIMPs and downregulation of MMPs would seem to be pro-fibrotic, the balance appears more complex, as broad elevation of circulating MMPs and TIMPs with high levels of MMP-8, MMP-9, and TIMP-1 are associated with severe fibrotic disease [27, 28].

As well as being both initiators and responders in the inflammatory phase of fibrosis, epithelial cells can also directly mediate fibrosis [1]. During epithelial–mesenchymal transition (EMT), key cytokines such as TGF-β, IL-4, and IL-13 can stimulate epithelial cells to detach from the basement membrane and differentiate into fibroblasts. This is signified by the loss of epithelial marker E-cadherin and expression of mesenchymal cell marker vimentin, and this process is a significant source of wound-healing fibroblasts. Fibroblasts release MMP-2, -3, and -9, which degrade the basement membrane and further recruit epithelial cells to undergo EMT. Failure to downregulate EMT further contributes to fibrosis [25, 29].

The multiple roles of epithelial cells in the initiation and development of fibrosis underline their importance. One of the crucial roles of epithelial cells in inflammation, wound healing, and fibrosis is the secretion of cytokines, especially the alarmin IL-33.

## IL-33 biology

Interleukin-33 (IL-33) is a cytokine of the IL-1 family, which also contains IL-1α, IL-1β, IL-18, IL-36, IL-37, and IL-38. As described above, many of the members of this family are implicated in the development of fibrosis. IL-33 holds an essential role in maintaining tissue homeostasis while directing adaptive immune responses to injury and environmental stress [30]. Its receptor ST2 is encoded by the *Il1rl1* gene, which is transcribed into two splice-variant proteins: a membrane-bound (ST2L) and a soluble form [soluble ST2 (sST2)] [31]. IL-33 is constitutively produced in the nucleus of endothelial and epithelial cells, and is released upon their necrosis, which is induced by viruses, allergens, and mechanical wounding [32]. Necrosis was long thought to be the only mode by which IL-33 could be released; however, recent data indicate that lower levels of IL-33 can also be released from viable cells via perforin 2 or gasdermin C pores in dendritic cells (DCs) and intestinal goblet cells, respectively [33, 34]. It is currently unknown which of these modes of IL-33 release is most important during fibrotic disease.

The IL-33 protein can be divided into three regions: a C-terminal IL-1-like cytokine domain, a central domain, and an N-terminal nuclear domain. The latter is characterized by a chromatin-binding motif, which tethers IL-33 in the cell nucleus by anchoring it to the histone H2A-H2B dimer. Full-length IL-33 is active, but a range of proteases can cleave the protein in its central domain, releasing the IL-1-like cytokine domain (‘mature IL-33’) that has >10-fold greater potency at stimulating ST2-expressing cells [35].

Retention of IL-33 within the nucleus prevents constitutive release: when the N-terminal chromatin-binding motif of IL-33 was deleted in transgenic mice, uncontrolled IL-33 release resulted in fatal ST2-dependent inflammation [36, 37]. Extracellular IL-33 can be blocked by sST2, while intracellular IL-33 can be cleaved in the IL-1-like domain by the apoptotic caspases 3 and 7, inactivating the cytokine [38]. Furthermore, on release, IL-33 rapidly (<30 min) forms two pairs of intramolecular disulphide bonds, rendering the cytokine inactive [14].

The IL-33 receptor is a heterodimer comprised of ST2, which binds directly to IL-33, and IL-1RAcP. IL-1RAcP is recruited to the IL-33-ST2 complex, juxtaposing cytoplasmic Toll/IL-1R domains and mediating signalling [39]. Signalling occurs after the myeloid differentiation factor 88 (MyD88), the adaptor protein TNF receptor-associated factor 6 (TRAF6), and the kinases IL-1R-associated kinase 1 (IRAK1) and IRAK4 are recruited, in turn activating the NF-κB and mitogen-activated protein kinases (ERK, p38 and JNK) pathways [40]. Several processes regulate ST2 signalling: ST2 can be phosphorylated and ubiquitylated to be internalized and degraded; ST2 and IL-1RAcP dimerization can be disrupted by the single immunoglobulin domain IL-1 receptor-related molecule (SIGIRR), and IL-33 can be sequestered by sST2 to prevent its signalling through ST2 on target cells [36, 41, 42]. Thus, the activity of IL-33 is closely regulated to prevent dangerous inflammatory responses.

## Cellular responses to IL-33

Murine and human ILC2s, CD4+ and CD8+ T cells, basophils, mast cells, eosinophils, DCs, monocytes, natural killer T cells, and murine natural killer cells have been found to express membrane-bound ST2. Moreover, ST2 has been found on stromal cells, including epithelial cells, endothelial cells, and fibroblasts, highlighting the role of IL-33 in varied contexts. While expression of ST2 is typically better characterized in mice compared with humans, especially for myeloid cells, some studies have identified constitutive ST2 expressed in human macrophages and DCs [43, 44].

IL-33 can induce cytokine secretion in several cell types: it acts on basophils to release IL-13, on ILC2s to produce IL-5 and IL-13, and on mast cells to secrete TGF-β [45, 46]. IL-33 also synergizes with IgE cross-linking to favour basophil and mast cell degranulation [47]. On eosinophils, IL-33 enhances adhesion, survival, and degranulation while inducing eosinophil IL-8 and superoxide anion release [48]. Moreover, IL-33 amplifies the IL-13-mediated polarization of M2 macrophages, particularly the M2a subtype, which mediate fibrogenesis via TGF-β production and downregulate inflammation via the secretion of IL-10 and arginase 1 [49–51]. IL-13 also induces the differentiation of fibroblasts into myofibroblasts, which release IL-33 along with collagen, providing an IL-33/IL-13 feed-forward loop which may further amplify pro-fibrotic responses [30, 52, 53]. IL-33 polarizes human and murine naive CD4+ T cells towards T helper 2 (Th2) differentiation while activating differentiated Th2 cells to produce IL-4, IL-5, and IL-13 in an antigen-independent manner [54, 55]. Therefore, IL-33 can promote and amplify many critical pro-fibrotic pathways, leading to the release of other pro-fibrotic mediators such as TGF-β and promoting ECM remodelling and fibrosis.

**Figure 1:**
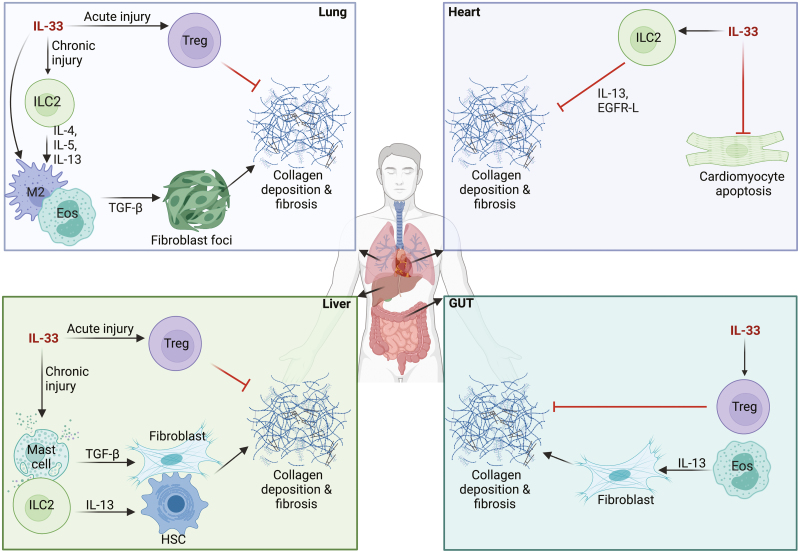
roles of IL-33 in organ fibrosis. In the lung, liver, and gastrointestinal tract, there is a delicate balance between the resolution of inflammation and fibrosis progression. During acute injury, IL-33 favours the resolution of inflammation by promoting regulatory T cell activity. However, in the presence of chronic injury, IL-33 induces a signalling and cell activation cascade, ultimately promoting fibroblast-mediated fibrinogenesis and fibrosis. Conversely, IL-33 covers cardioprotective functions in the heart by preventing cardiomyocyte apoptosis and fibrosis. IL, interleukin; Treg, T regulatory cell; ILC2, innate lymphoid cell of type 2; M2, type 2 macrophage; TGF-β, transforming growth factor β.

Dependent on the context, IL-33 can have both pro-inflammatory and anti-inflammatory effects. Regulatory T cells (T_regs_) express ST2, and IL-33-expressing DCs can stimulate T_regs_ via ST2, activating them and controlling allergic and inflammatory immune responses [33]. Therefore, due to the varied roles of IL-33 in fibrosis and the multitude of cells it can target, it is imperative to understand the specific role that IL-33 plays in the cellular milieu of each organ during fibrosis (Fig. 1).

## IL-33 in lung fibrosis

The most common and severe form of interstitial lung disease is idiopathic pulmonary fibrosis (IPF) [56]. IPF is a progressive, intractable lung scarring disease with a poor long-term prognosis. Notably, an estimated 32,500 people are affected by the disease in the UK, with more than 9000 new diagnoses and 5300 people dying of IPF each year [57].

The aetiology of IPF is still unknown, although there is an element of (highly polygenic) genetic susceptibility with IPF development, implicating aberrant expression of multiple genes, including those involved in cellular proliferation such as DEPTOR, KIF15, and MAD1L1 [58]. Environmental risk factors, including exposure to tobacco smoke, metal and wood dust, and gastroesophageal reflux have also been implicated [59–61]. These factors lead to bronchial and alveolar epithelial injuries, with consequent release of IL-33, activation of fibroblasts, and uncontrolled tissue repair mechanisms involving the remodelling of the ECM [62]. Hallmark histological changes observed in IPF-affected lungs are the presence of fibroblast foci, honeycombing and or disruption of the lung architecture with a patchy lung involvement by fibrosis, indicating focal points of damage and subsequent scarring [63]. Notably, human BAL samples and lung tissues of IPF-affected patients showed increased IL-33 mRNA and protein levels, markers associated with a response to damage [64–66].

During IPF, activation of ILC2 cells by IL-33 leads to eosinophil recruitment and M2 macrophage polarization while IL-33 directly signals on these cells to induce further TGF-β secretion, which activates fibroblasts [64]. Notably, IL-33 can act as a gene regulator in the lung by amplifying an IL-13-driven polarization of macrophages towards the M2 phenotype [64]. Indeed, knocking out the genes Akt1 and Akt2 in macrophages exposed to IL-33 resulted in reduced IL-13 and TGF-β production and impaired fibrinogenesis, indicating that these genes are essential for IPF progression in response to IL-33 [67, 68]. It has also been recently hypothesized that IL-33 can affect fibroblast differentiation and activation directly within the lung, given the expression of surface ST2 by lung fibroblasts [69, 70].

A well-characterized animal model of IPF involves the administration of bleomycin sulphate (BLM), an anti-cancer drug which can also cause lung fibrosis in humans [41, 71]. BLM induces reversible lung fibrosis in mice, whereby pathology is characterized by an early inflammatory phase and a subsequent fibroproliferative phase. The inflammatory phase is associated with injury to epithelial cells and leucocyte infiltration, while the fibroproliferative phase is associated with ECM remodelling and fibrosis. Several studies have demonstrated that IL-33- and IL-33-positive cells are elevated during the inflammatory and pro-fibrotic stages [41, 65, 71–73]. Bleomycin can also favour the secretion of IL-33 by macrophages and epithelial cells in an ST2- and Th2-independent manner, with IL-33 possibly changing the gene expression of several cytokines such as IL-6, MCP-1, TGF-β, and the heat shock protein 70 in the nucleus of IL-33-expressing cells [53, 65, 74].

Several studies have investigated IL-33 as a target for intervention in the BLM model of fibrosis. Genetic ablation of ST2, administration of anti-IL-33, or lentiviral overexpression of the sST2 decoy receptor during the inflammatory phase of the BLM model attenuated IL-13, IL-33, and TGF-β expression while halting pulmonary fibrosis and improving survival rates in BLM-treated mice [64, 73]. Conversely, intratracheal instillation of IL-33 in BLM-treated mice increased pro-fibrotic collagen production [65].

However, due to the pleiotropic effects of IL-33, IL-33 blockade in fibrosis could also have disadvantageous effects. IL-33 administration increased survival, while IL-33 deficiency decreased survival during BLM-induced acute lung injury due to its impact on ST2+ T_regs_. IL-33 induced T_reg_ IL-13 production, limiting the inflammatory response [46]. Therefore, the timing and context of IL-33 blockade are critical in pulmonary fibrosis, and in the early inflammatory phase, IL-33 may be tissue protective.

In established fibrotic disease, most evidence points to a pro-fibrotic role of IL-33, inducing differentiation and activation of collagen-secreting fibroblasts in mice and humans. Therefore, IL-33 is a potential target for treatment against pulmonary fibrosis.

## IL-33 in hepatic fibrosis

Hepatic fibrosis was initially considered to be an irreversible disease (like IPF), damaging the hepatic parenchyma and causing its collapse [75, 76]. However, later studies demonstrated that even advanced hepatic fibrosis, but not cirrhosis, is a reversible process [77]. Hepatic fibrosis results from sustained liver injury caused by multiple factors, including alcohol abuse, fat deposition, and hepatotropic viral infections [78]. Following acute injury, apoptotic or necrotic hepatocytes are replaced by new parenchymal cells [79]. However, chronic injuries eventually lead to the failure of the wound-healing response and the replacement of hepatocytes by the ECM, which is abundant in fibrillar collagen [80].

In the liver, hepatic stellate cells (HSCs) or hepatocytes are the primary source of IL-33 [45, 81, 82]. HSCs can transition and differentiate from quiescence to ST2+ myofibroblasts via IL-13, which is secreted by immune cells following IL-33 signalling [81, 82]. During acute injury to the liver, the release of IL-33 by damaged hepatocytes promotes tissue healing, while in the case of chronic injury, the IL-33 acts as a critical fibrotic player [81]. Indeed, treatment with recombinant IL-33 (rIL-33)-attenuated pathology in a mouse model of steatosis-related non-alcoholic fatty liver disease at the cost of increasing ST2-dependent fibrosis of the liver, thus indicating that IL-33 can have both protective and deleterious pro-fibrotic roles in regulating liver homeostasis [73, 83].

During hepatic fibrosis, IL-33 and ST2 mRNA and protein levels were increased in murine and human fibrotic livers compared to healthy controls [83, 84]. In the liver, IL-33 induced the secretion of IL-13 from ILC2s, which in turn increased the differentiation and activation of HSCs by either directly acting on the HSCs or by enhancing IL-6 and TGF-β signalling, inducing HSC secretion of fibrillar collagen, causing fibrosis [36, 70]. ST2-deficient mice are protected from liver inflammation and fibrosis in models of high-fat diet-induced obesity and after administration of the pro-fibrotic agent carbon tetrachloride [83, 84].

Serum sST2 concentration directly correlates with disease severity and progression in hepatic fibrosis patients, making it a useful biomarker. This further highlights the importance of the IL-33 pathway in hepatic fibrotic disease [85–87]. As well as its role in promoting fibrosis in chronic liver injury, IL-33 can also serve a protective role during acute inflammation. Indeed, similarly to its role in the lung, IL-33 promotes the expansion and survival of ST2+ T_regs_ within the hepatic adipose tissue, attenuating inflammation during acute injury [45, 88, 89]

Therefore, these findings suggest that IL-33 may be essential to activate protective mechanisms during acute inflammation. However, IL-33 could also be a leading pro-fibrotic agent during chronic inflammation, inducing ECM release in an ST2- and Th2-dependent manner.

## IL-33 in gastrointestinal fibrosis

IL-33 has been implicated in chronic inflammatory diseases of the gastrointestinal tract where fibrosis has a pathogenic role, such as eosinophilic oesophagitis (EoE) and inflammatory bowel disease (IBD).

### Eosinophilic oesophagitis

Patients with EoE present inflammatory responses in the oesophagus thought to be caused by environmental factors such as food antigens, aeroallergens, and an altered microbiome. Clinically, patients show symptoms of food bolus obstruction and recurrent dysphagia when eating. The underlying pathophysiology is not fully understood [16]. From what is known, the wound-healing response is activated in response to environmental factors, culminating in the activation of fibroblasts. Fibroblasts then contribute to the formation of strictures in severe cases of EoE, leading to the dysphagia seen in EoE-affected patients [12]. As the name suggests, eosinophils dominate cellular infiltrates in EoE, and EoE is often associated with the development of other allergic diseases such as atopic dermatitis and asthma [16, 90]. Therefore, EoE pathology is an example of the close links between allergic and fibrotic disease.

ST2 expression is increased in paediatric EoE biopsies compared with controls, and those individuals reporting atopic dermatitis alongside EoE showed further increased ST2 expression [90]. Flow cytometry analysis revealed increased levels of ST2 expression on oesophageal eosinophils, mast cells, and Th2 cells compared with their blood-derived counterparts in both healthy and EoE-affected subjects [91]. Epithelial cells from active EoE human biopsies demonstrate increased protein expression of nuclear IL-33, normalizing only upon disease remission. There is also a positive correlation between eosinophil counts and ST2 mRNA expression in EoE, suggesting a contribution of the IL-33/ST2 axis to EoE pathogenesis [90].

In a mouse model of EoE, where animals are epicutaneously sensitized to ovalbumin (OVA) protein and then challenged with intranasal OVA, pathology could be abrogated by genetic ablation of ST2 or administration of anti-ST2 monoclonal antibody (mAb). In this model, eosinophilic inflammation was drastically reduced on basophil depletion or when ST2 deficiency was limited only to basophils [90]. Therefore, basophils appear critical to this axis.

In EoE, the IL-33 pathway appears to be firmly associated with disease progression; therefore, this may be an ideal target for intervention.

### Inflammatory bowel disease

IBD includes Crohn’s disease and ulcerative colitis. These conditions develop in genetically susceptible individuals leading to chronic inflammation in the gut with resultant damage to gastrointestinal tissues [92]. The trigger is unknown, and the pathogenesis is multifactorial with microbial dysbiosis, altered epithelial barrier permeability, and persistent inflammatory response in the underlying mucosa [93]. Chronic inflammation and aberrant wound healing cause fibrosis and strictures in approximately 30% of Crohn’s disease patients, mainly in the small bowel [94]. Stricturing is much less common in UC, affecting <5% of UC patients. There are no preventative treatments, and the resultant gut obstruction requires surgery [95].

Genetic polymorphisms in the IL-33 gene correlate strongly with IBD susceptibility, and carriage of the risk allele is associated with a severe disease phenotype [96]. In human IBD, sST2 expression is higher in UC (but not Crohn’s) intestinal biopsies, with increased levels of serum sST2 in UC when compared with healthy controls [97]. Gene expression of IL-33 was also upregulated in human colonic tissues from IBD patients and in murine dextran sodium sulphate (DSS) model of colitis. IL-33 aids the restoration of goblet cell function following the characteristic depletion seen in gut inflammation in the DSS-induced murine colitis model, suggesting a protective role against colitis [98]. Protective effects of IL-33 have been further corroborated in the DSS murine model by demonstrating that IL-33/ST2 signalling induces microRNA-320 in epithelial tissue, promoting epithelial repair and resolving inflammation [99].

Treatment with anti-ST2 mAb, or genetic ablation of ST2, significantly reduces pathology in the DSS model of colitis. Furthermore, IL-33 was also shown to negatively affect intestinal barrier permeability in human intestinal monolayer cultures and in a murine *in vivo* model [100]. ST2 knockout mice showed faster regeneration of the intestinal epithelium following biopsy-induced injury, indicating that the IL-33/ST2 axis may have a role in delaying the wound-healing response [100]. In the DSS model using IL-33 knockout mice, IL-33 has been linked to delayed tissue damage resolution, again demonstrating a detrimental effect in IBD [101]. In a human paediatric study of stricturing Crohn’s, eosinophils were found in the mucosal layer of inflamed, treatment-naive ileal tissue. Furthermore, human eosinophils were activated by IL-33, resulting in their degranulation and IL-13 expression. In co-culture with intestinal fibroblasts, IL-33-activated eosinophils led to increased expression of inflammatory cytokines TNF-α, IL-1β, and IL-6 in addition to eosinophil-targeting chemoattractants Eotaxin-2 and -3, providing evidence for an IL-33-eosinophil-fibroblast pathway in stricturing Crohn’s disease [102, 103]. These results implicate an inflammatory role of IL-33 in colitis. Conversely, IL-33 signalling has been shown to enhance TGF-β-induced differentiation of T_reg_ cells, their recruitment to inflamed tissue, thus constraining inflammation in IBD [104].

It is not clear if IL-33 has a detrimental or protective role in IBD, IL-33, and its receptors are expressed in IBD tissue, but the biological consequences of this are not fully understood. Therefore, further work in human gastrointestinal models is necessary to fully understand the mechanisms by which IL-33 modulates fibrosis in IBD.

## IL-33 in skin fibrosis

IL-33 has been implicated in the skin fibrosis seen in systemic sclerosis (SSc), an autoimmune fibrotic disease of the skin, internal organs, and blood vessels, which has the highest mortality of the rheumatic diseases [105]. In human SSc, serum IL-33 was elevated compared with healthy control serum, and serum IL-33 correlated with severity of disease [106, 107]. Likewise, in a murine model of skin fibrosis induced by subcutaneous bleomycin injection, IL-33 was elevated in the dermis. In the mouse model, deletion of ST2 caused a significant increase in bleomycin-induced fibrosis, implying a protective role for IL-33 in this model, thought to be via T_reg_ activation [106]. In contrast, in a mouse model of skin wounding, IL-33 was also upregulated at the site of healing and subsequent scarring, and administration of recombinant IL-33 could induce scar formation in a normally non-scarring fetal model [108]. Therefore, during skin healing and scarring, IL-33 may act at early time points to induce healing (but also scarring and fibrosis), while at later, chronic time points, IL-33 may have a more pro-resolution role. This conflicting evidence is similar to that seen in gastrointestinal fibrosis, and further investigations into its role in skin fibrosis is required to determine under which situations IL-33 is pro-or anti-fibrotic.

## IL-33 in renal fibrosis

Renal fibrosis can occur as a result of multiple chronic kidney diseases, including chronic kidney transplant rejection, and is an indicator of kidney failure. In chronic kidney transplant rejection, serum and kidney IL-33 levels are increased compared to healthy controls, and *in vitro* IL-33 promotes EMT of tubular epithelial cells, podocytes and fibrocytes into mesenchymal fibroblasts able to secrete collagen and promote fibrosis. Similarly, fibroblastic mesangial cells can be activated and promote fibrosis at the glomerulus [109, 110].

In mouse models of kidney fibrosis, IL-33 has been shown to have both pro- and anti-fibrotic effects. In the unilateral urinary obstruction mouse model, IL-33 or ST2 deficiency reduced kidney injury and fibrosis [111], while in a mouse model of ischaemia reperfusion injury (IRI), blocking IL-33 with sST2 resulted in reduced pro-fibrotic myofibroblast accumulation [112, 113]. However, following IRI resolution, administration of IL-33 resulted in protective roles promoting T_reg_ differentiation and M2 macrophage polarization [113].

These findings suggest that IL-33 might have protective roles during acute kidney injury that could reverse to deleterious pro-fibrotic functions in the presence of chronic renal pathology, and further underline the role of IL-33 in acute versus chronic responses.

## IL-33 in cardiac fibrosis

Cardiovascular disease is the leading cause of death worldwide, accounting for 17.9 million global deaths, of which 85% were attributed to heart attack and stroke [114]. Cardiovascular fibrosis occurs following damage to the cardiac tissue. As cardiomyocytes are mostly unable to regenerate following injury, tissue remodelling occurs, and scar tissue is formed to compensate for the necrosed cells and prevent rupture of the ventricular wall [115, 116]. In severe cases, ventricles elongate to increase their volume and maintain cardiac function, but persistence will decrease cardiac output and lead to heart failure [117]. Following cardiac injury, granulocytes, monocytes, leukocytes, and DCs are recruited [117]. Cytokine signalling leads to the activation and differentiation of tissue-resident fibroblasts into myofibroblasts, stabilizing the scar, and secreting ECM proteins to fill the injured area [118].

Notably, ST2 has been identified as a biomarker for heart failure and myocardial infarction, leading to investigations of its role in cardiac pathophysiology [119].

Cardiac fibrosis is a common occurrence in heart disease due to mechanical overload, and IL-33 and ST2 expression are upregulated in rat cardiac fibroblasts subjected to cyclic mechanical strain. This is a protective pathway, as rIL-33 administration to mice following transverse aortic constrictive surgery improved survival, while ST2-deficient mice showed enhanced cardiac fibrosis [120]. Furthermore, IL-33 prevents apoptosis of cardiomyocytes in mice following ischemia/reperfusion injury through upregulation of the anti-apoptotic proteins cIAP (cellular inhibitor of apoptosis proteins 1), XIAP (X-linked inhibitor of apoptosis protein), survivin, Bcl-2 (B-cell lymphoma 2), and Bcl-xL (B-cell lymphoma-extra large), reducing the infarct and fibrosis volume 15 days post-reperfusion. IL-33 also increased the gene expression of IL-10 and IL-4 and decreased the Type 1 inflammatory cytokine IFN-γ in cardiac tissues [121].

Further protective actions of IL-33 were demonstrated by administration of IL-33 in the isoproterenol (ISO)-induced cardiac injury model, showing that IL-33 activated cardiac ILC2s, which through release of IL-13 and epidermal growth factor receptor ligands protected against cardiac fibrosis, resolving ISO-generated changes in ECM-associated gene expression, and decreasing the fibrotic lesion size [122].

Therefore, in the heart, the IL-33 pathway appears to have a dominantly protective effect against fibrotic disease. The differences between IL-33 effects in fibrotic cardiac disease and fibrosis in other organs may stem from the necessity for scar tissue formation to compensate for the poor regenerative properties of cardiomyocytes. However, phenotyping of cardiac epithelial and immune cells in comparison with other fibrotic diseases could elucidate differences in outcome including differences in IL-33/ST2 expression.

## Conclusion

IL-33 has clear effects on inflammation and fibrosis in multiple diseases, tissues, and contexts. Due to the complexity of the response to IL-33, it can have both protective and detrimental effects in the same organ system. Timing and microenvironmental context appear to be key. The balance between anti- and pro-fibrotic activity is not well characterized in any organ type, and elucidation of circumstances under which IL-33 becomes pro-fibrotic could provide valuable clinical insight into targeting this pathway for therapeutic effects.

It should be noted that while IL-33 has important roles in fibrotic disease, it does not act in isolation: IL-25 and TSLP, like IL-33, are released from the epithelium during type 2 immune responses and damage, and these three cytokines may act in concert during wound healing and fibrosis. In models of asthma and parasitic infections, blocking any of IL-25, IL-33, or TSLP alone showed only modest results, while combinatorial targeting of IL-33, IL-25 and TSLP significantly reduced collagen expression and fibrosis [123]. Therefore, IL-25 and TSLP should also be taken into consideration to better understand the pro-fibrotic functions of IL-33.

Moreover, the use of translational systems not only involving animal models, but also human-derived settings could help further elucidate this delicate balance between protection and fibrinogenesis exerted by IL-33. One of such approaches could involve the use of human organoid cultures which provide faster and more robust outcomes while accurately representing human tissues and providing a larger quantity of material to work with than animal models do. Indeed, recent studies demonstrated organoids to be successful in representing pro-fibrotic pathology of the lung, liver, and gastrointestinal tract [124–126].

In order to develop therapeutic strategies against IL-33, clinical trials are required to test potential IL-33-targetted treatments in patients. Currently, clinical trials are assessing the efficacy of anti-IL-33 monoclonal antibody treatments (Itepekimab, Etokimab and Tozorakimab) in chronic obstructive pulmonary disease, asthma, atopic dermatitis and peanut allergy [127–130]. Although these have shown efficacy in reducing inflammation, they have yet to be investigated in lung fibrosis, where they may also have therapeutic potential [131].

In other fibrotic diseases, IL-33 blockade could be helpful or deleterious, depending on tissue site, immune environment and fibrotic mechanisms. Only by better understanding how these diseases develop, and the role for IL-33 in this process, can we predict the effects of IL-33 modulation.

## Abbreviations

Bcl-2: B-cell lymphoma 2
Bcl-xL: B-cell lymphoma-extra large
CD: Crohn’s disease
cIAP: Cellular Inhibitor of Apoptosis Protein 1
COPD: chronic obstructive pulmonary disease
DAMPs: damage-associated molecular patterns
DC: dendritic cell
DSS: dextran sodium sulphate
ECM: extracellular matrix
EGFR: epidermal growth factor receptor
EMT: epithelial–mesenchymal transition
EoE: eosinophilic oesophagitis
HSC: hepatic stellate cell
IBD: inflammatory bowel disease
IFN-γ: interferon gamma
IL: interleukin
ILC2: group 2 innate lymphoid cells
LM: bleomycin
IPF: idiopathic pulmonary fibrosis
IRI: ischaemia reperfusion injury
ISO: isoproterenol
mAb: monoclonal antibody
miR-320: microRNA-320
MMP: matrix metalloprotease
NF-kB: nuclear factor kappa-light-chain-enhancer of activated B cells
OVA: Ovalbumin
PDGF: platelet-derived growth factor
rIL: recombinant interleukin
SSc: systemic sclerosis
sST2: soluble ST2
TGF-β: transforming growth factor beta
Th2: T helper 2
TIMP: tissue inhibitors of metalloproteinases
TNF-α: tumour necrosis factor alpha
T_reg_: T regulatory cell
TSLP: thymic stromal lymphopoietin
UC: ulcerative colitis
UUO: unilateral urinary obstruction
XIAP: X-linked inhibitor of apoptosis protein
α-SMA: α-smooth muscle actin
